# Maize Crops Under Rising Temperatures: Bacterial Influence on Biochemical and Lipidomic Changes Induced by Heat

**DOI:** 10.3390/plants14162593

**Published:** 2025-08-20

**Authors:** Ricardo Pinto, Paulo Cardoso, Bruno Carneiro, Glória Pinto, Carmen Bedia, Etelvina Figueira

**Affiliations:** 1 CESAM—Centre for Environmental and Marine Studies and Department of Biology, University of Aveiro, 3810-193 Aveiro, Portugal; rl.pinto@ua.pt (R.P.); gpinto@ua.pt (G.P.); 2 Department of Biology, University of Aveiro, 3810-193 Aveiro, Portugal; brunomcarneiro@ua.pt; 3 Department of Environmental Chemistry, Institute of Environmental Assessment and Water Research (IDAEA-CSIC), c/Jordi Girona 18–24, 08034 Barcelona, Spain; carmen.bedia@cid.csic.es

**Keywords:** thermotolerance, plant–microbe interactions, PGPB, sustainable agriculture, heat stress, biochemical status, lipid profile

## Abstract

Rising global temperatures are increasingly affecting plant performance, leading to reduced growth, altered metabolism, and compromised membrane integrity. Although plant growth-promoting bacteria (PGPB) show promise in enhancing thermotolerance, the underlying mechanisms remain insufficiently explored. Therefore, this study investigated the effects of PGPB inoculation on *Zea mays* under control (26 °C) and heat stress (36 °C) conditions. Maize plants were inoculated with two thermotolerant bacterial strains and their effects were compared to non-inoculated plants through morphometric, biochemical, and lipidomic analyses. Heat stress negatively affected germination (−35.9%), increased oxidative stress (+46% for LPO, +57% for SOD, +68% for GPx), and altered leaf lipid composition, particularly fatty acids, glycerolipids, and sphingolipids. Inoculation with *Pantoea* sp. improved germination by 15% for seeds exposed to heat stress, increased growth (+28% shoot and +17% root), enhanced antioxidant defenses (+35% for CAT and +38% for APx), and reduced membrane damage by 65% compared with the control. Lipidomic profiling revealed that inoculation mitigated temperature-induced lipid alterations by reducing triacylglycerol accumulation and preserving the levels of polyunsaturated galactolipids and hexosylceramides. Notably, *Pantoea* sp.-inoculated plants under heat stress exhibited lipid profiles that were more similar to those of control plants, suggesting enhanced heat resilience. These results underscore the importance of specific plant–microbe interactions in mitigating heat stress and highlight PGPB inoculation as a promising strategy to enhance crop performance and resilience under projected climate warming scenarios.

## 1. Introduction

Maize (*Zea mays*) can be grown in a wide array of agroclimatic conditions, including diverse temperatures, altitudes, latitudes, and land and soil types. Its high yields compared to those of other cereals make it particularly attractive in areas with land scarcity and high population pressure [1]. Thus, it is not surprising that in 2021, maize was the second-most-produced agricultural crop worldwide (1.2 billion tons of kernels), revealing its economic and social importance [2].

The global population is expected to increase, imposing pressure on food production. Different models of climate change generally describe an increase in planetary surface temperature [3]. This increase will affect species, ecosystem functioning, and agricultural production. When exposed to prolonged periods of elevated temperatures, maize plants can experience a range of negative effects on grain maturation and yield [4]. Few studies have addressed the isolated effect of heat stress in the early growth phase [5] and prioritize the effect of temperature on plant production and maturation. High temperatures inhibit photosynthetic processes in plants, leading to a decrease in plant energy production and growth [6].

Many studies on the response of maize to climate change do not isolate the effect of temperature from drought, as plant metabolic and molecular responses to the combined effect of heat and drought are unique and cannot be extrapolated from the responses induced by each of these stresses applied individually [7]. In natural systems, these two stresses can be associated (such as the Mediterranean climate), and it is important to evaluate their combined effect to predict the resilience or sensitivity of systems in a changing climate. However, there are climates where drought and high temperature are not synchronous, and irrigation can also reduce, or even eliminate, drought from agricultural fields, retaining the effect of temperature stress.

In fact, when water in the soil and air humidity are maintained, plants can more easily deal with increased temperatures. The flow of water from the roots to the leaves maintains evapotranspiration, a process that effectively cools the leaves and increases plant tolerance to heat stress [8].

Heat stress can lead to oxidative stress in plant cells, which can result in lipid peroxidation, affecting cell membrane integrity, and protein carbonylation, which can affect enzyme activity. Plant cells scavenge reactive oxygen species’ (ROS) effects by enhancing antioxidant enzyme activities such as superoxide dismutase, catalase, glutathione S-transferases, glutathione, and ascorbate peroxidases [9].

Plant membrane lipids play a crucial role in maintaining the structural integrity and functionality of cell membranes [10]. The main types of lipids found in plant membranes are phospholipids, glycolipids, and sterols. The composition and arrangement of these lipids can influence membrane fluidity, flexibility, and permeability [10]. Plants can adjust membrane lipid composition in response to environmental factors, including temperature changes [10]. Heat stress can increase membrane fluidity, making the lipid bilayer more permeable [10]. This can affect the selective transport of ions and molecules across the membranes. Cell response mechanisms may lead to changes in the fatty acid composition of membrane lipids, producing lipids with shorter and more unsaturated fatty acid chains [11].

To mitigate the effects of high temperatures on maize, farmers and researchers can employ various strategies, such as sowing heat-tolerant varieties, adjusting agricultural practices (e.g., irrigation and sowing time), or applying plant growth-promoting bacteria (PGPB). Inoculation with PGPB has emerged as a promising methodology for coping with temperature stress [12]. PGPB can be found in the rhizosphere and can establish mutually beneficial relationships with plants. They promote plant growth and development through several mechanisms, including nutrient solubilization and uptake, hormone production, increased stress tolerance, biocontrol activity, and soil structure improvement [13]. During heat stress, PGPB can enhance nutrient and water uptake, helping plants maintain their metabolic processes even at high temperatures. Some PGPB produce plant hormones [14], whereas others may synthesize osmoprotectants, such as proline, that help plants maintain cellular hydration [15], avoiding protein precipitation. Moreover, PGPB can protect the photosynthetic apparatus through the production of antioxidant compounds or enzymes that help plants cope with oxidative stress [16]. Bacteria can also influence the lipid profile of plants [17]. Overall, some PGPB can trigger plant innate defense mechanisms, leading to the induction of systemic resistance against stress factors [18]. However, many of these effects have been reported in plants subjected to the combined effects of water and heat stress, and the isolated effects of temperature are poorly understood.

PGPB inoculation has gained attention as a sustainable and eco-friendly method for enhancing crop productivity. However, there is a lack of understanding of how plant–bacteria interactions change in high-temperature scenarios. This study aimed to provide novel information to increase the knowledge about the effect of bacterial inoculants on plant tolerance to heat stress. Maize plants were exposed to two temperatures (control and high) and different inoculation conditions (no inoculation and inoculation with two rhizobacterial strains tolerant to temperature). The bacterial strains used in this study were obtained from two agricultural soils in Vila do Conde and Mogadouro, northern mainland Portugal. Two strains (*Herbaspirillum* sp. A8S23D6 and *Pantoea* sp. H11S28N2) exhibited higher growth and plant growth promotion abilities (indole-3-acetic acid, siderophores, volatile organic compound production, phosphate solubilization, and antifungal activity) at 40 °C compared to the control temperature (26 °C) (unpublished). This approach allowed us to achieve the following: (1) establish the effect of temperature on maize plants under the growth conditions used; (2) determine the plant promotion ability of two thermotolerant strains (*Herbaspirillum* sp. and *Pantoea* sp.) at the control temperature (26 °C); and (3) identify the protection afforded to heat-stressed maize plants by the two bacterial strains. These effects were estimated using morphometric, biochemical, and lipidomic parameters.

## 2. Results

### 2.1. Seed Germination and Plant Growth

Seed germination was affected by temperature and inoculation (Table S1). Temperature significantly decreased seed germination under all conditions. At 26 °C, only *Herbaspirillum* sp. showed increased germination, but at 36 °C, both strains showed increased germination rates. At the end of the experiment, colony-forming unit (CFU) determination confirmed that the temperature did not affect bacterial survival in the soil during the plant growth period. The number of viable cells was identical between both strains for 26 °C, but at 36 °C viable cells in the soil inoculated with *Pantoea* sp. were higher than with *Herbaspirillum* sp. (Table S2).

**Figure 1:**
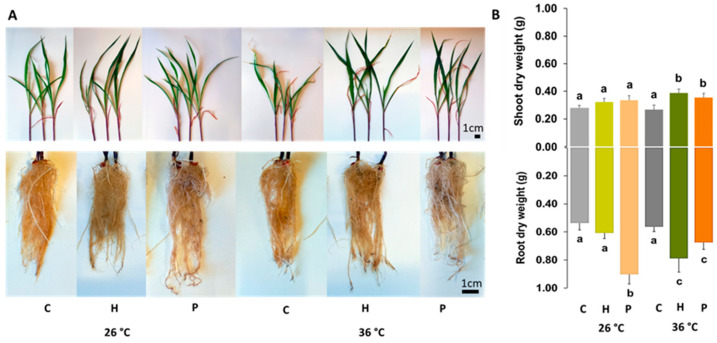
Growth of *Zea mays* was influenced by temperature (26 °C and 36 °C) and inoculation (Control—C, *Herbaspirillum* sp.—H, *Pantoea* sp.—P). (**A**) Shoot and root lengths and morphology. (**B**) Shoot and root dry weights. Data are presented as the mean ± SD (n = 5). Significant differences (*p* < 0.05) between the conditions are represented by lowercase letters.

Temperature did not significantly change shoot and root dry weights in the non-inoculated plants. At 26 °C, both strains increased shoot and root growth (Figure 1A,B). However, the only significant difference was observed in the roots of the plants inoculated with *Pantoea* sp. (P), with a 68% increase compared to the control. At 36 °C, both bacteria significantly increased the shoot and root dry weights. Inoculation with *Herbaspirillum* sp. induced greater differences (45% and 40% for roots and shoots, respectively).

The interaction of both factors (temperature and inoculation) was significant for shoots and roots, with the effect of inoculation increasing at higher temperatures (Table S3).

### 2.2. Biochemical Analysis

Plant biochemistry was influenced by growth conditions. Biomarkers related to cell functioning (electron transport system, proteins, photosynthetic pigments, and energy reserves), osmolytes (proline and sugars), oxidative stress (antioxidant enzymes), and cell damage (lipid peroxidation and protein carbonylation) were compared between conditions. Each factor was analyzed separately, and the interaction of the factors was also analyzed (Table S3) to understand the impact of each condition on plant biochemistry.

#### 2.2.1. Cell Functioning

**Figure 2:**
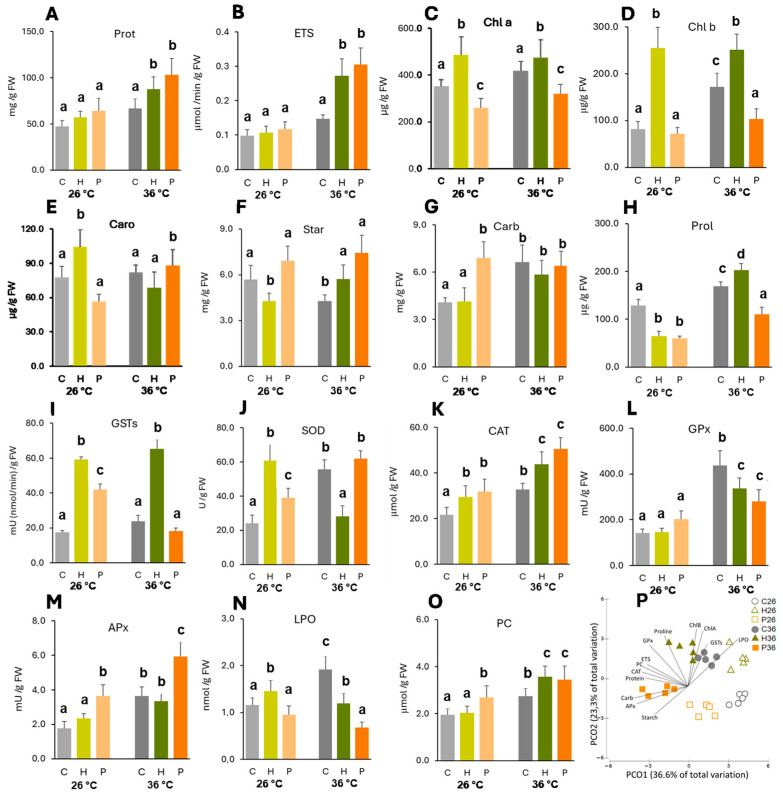
Biochemical parameters of *Zea mays* shoots influenced by temperature (26 °C and 36 °C) and inoculation (Control—C, *Herbaspirillum* sp.—H, *Pantoea* sp.—P). (**A**) Soluble protein content (Prot); (**B**) electron transport system activity (ETS); (**C**) chlorophyll a (Chl a); (**D**) chlorophyll b (Chl b); (**E**) carotenoids (Caro); (**F**) starch (Star); (**G**) soluble carbohydrates (Carb); and (**H**) proline (Prol). (**I**) Glutathione S-transferases (GSTs); (**J**) superoxide dismutase (SOD); (**K**) catalase (CAT); (**L**) glutathione peroxidase (GPx); (**M**) ascorbate peroxidase (APx) activity; (**N**) lipid peroxidation (LPO); (**O**) protein carbonylation (PC); (**P**) Principal Coordinates Ordination (PCO) based on the biochemical parameters of *Zea mays* shoots under different inoculation and temperature conditions. Data are presented as the mean ± SD (n = 5). Significant differences (*p* < 0.05) between the conditions are represented by lowercase letters.

Temperature had a slight impact on the cell functioning of non-inoculated plants, as for most parameters, no significant differences were observed. The only exception was chlorophyll b (Figure 2D), which significantly increased in plants exposed to 36 °C.

At 26 °C, both strains were able to increase, but not significantly, protein content and electron transport system (ETS) activity (Figure 2A,B). Inoculation with *Herbaspirillum* sp. significantly increased all photosynthetic pigments (Figure 2C–E), especially chlorophyll b (2.1-fold increase), and decreased starch concentration (Figure 2F).

At 36 °C, the two strains showed significantly increased protein and ETS activity. *Pantoea* sp. induced the greatest increase (50% protein content and 100% ETS). *Herbaspirillum* sp. evidenced a trend of significantly increasing chlorophylls and starch (chlorophyll b and starch, respectively). *Pantoea* sp. significantly reduced chlorophylls a and b and increased the starch content (Figure 2C,D,F). ETS, chlorophyll b, carotenoids, and starch showed significant interactions between the temperature and inoculation (Table S3).

#### 2.2.2. Osmolytes

Temperature induced significant increases in soluble carbohydrate and proline contents in non-inoculated plants (Figure 2G,H). In plants inoculated with *Herbaspirillum* sp., temperature also significantly increased both the parameters. Temperature did not influence the soluble carbohydrate content in plants inoculated with *Pantoea* sp.; however, proline levels were significantly higher at 36 °C.

At 26 °C, only *Pantoea* sp. was able to significantly increase (69%) soluble carbohydrate content (Figure 2G), and both strains significantly decreased proline levels compared to the control plants (Figure 2H).

At 36 °C, *Herbaspirillum* sp. and *Pantoea* sp. did not change soluble carbohydrates, but proline concentration was significantly increased by *Herbaspirillum* sp. and significantly decreased by *Pantoea* sp. compared with non-inoculated plants at 36 °C.

Interaction effects between temperature and inoculation were observed for soluble carbohydrates and especially for proline, where inoculation decreased proline at 26 °C and increased (*Herbaspirillum* sp.) or reduced (*Pantoea* sp.) proline at 36 °C (Table S3).

#### 2.2.3. Activity of Antioxidant Enzymes

Temperature significantly increased the activity of most antioxidant enzymes in the inoculated and non-inoculated plants, except for glutathione S-transferases (GSTs) in plants inoculated with *Pantoea* sp. and superoxide dismutase (SOD) in plants inoculated with *Herbaspirillum* sp.

At 26 °C, *Herbaspirillum* sp. was able to significantly increase GST (2.4-fold), SOD (1.5-fold), and catalase (CAT) (0.4-fold) activity (Figure 2I–K), and *Pantoea* sp. significantly induced the activity of most enzymes compared to control plants, except glutathione peroxidase (GPx) (Figure 2L).

At 36 °C, *Herbaspirillum* sp. significantly increased GSTs (1.8-fold) and CAT (0.3-fold) (Figure 2I,K), and *Pantoea* sp. significantly increased CAT (0.5-fold) and ascorbate peroxidase (APx) (0.6-fold) compared to non-inoculated plants at 36 °C (Figure 2K,M).

Except for catalase, for which the increase trend in enzymatic activity induced by inoculation was not changed by temperature, both factors interacted in determining the activity of antioxidant enzymes (Table S3).

#### 2.2.4. Cell Damage

Temperature significantly increased the membrane damage (LPO) of non-inoculated plants, but did not change LPO in inoculated plants (Figure 2N). Protein damage (PC) increased with temperature in non-inoculated and *Herbaspirillum* sp.-inoculated plants but was not significantly affected in plants inoculated with *Pantoea* sp. (Figure 2O).

At 26 °C, *Herbaspirillum* sp. increased and *Pantoea* sp. decreased (not significantly) LPO levels compared to non-inoculated plants, but significant differences between both bacteria were observed (Figure 2N). *Herbaspirillum* sp. did not induce changes in protein carbonylation, but *Pantoea* sp. showed a significant increase compared to the control plants (Figure 2O).

At 36 °C, both bacteria induced significant decreases in LPO (especially *Pantoea* sp.) and increased protein carbonylation compared to non-inoculated plants at 36 °C.

Interaction between temperature and inoculation was observed for LPO, with inoculation decreasing membrane damage at higher temperatures. Interaction between both factors was not observed for protein carbonylation (Table S3).

### 2.3. Multivariate Analysis of the Biochemical Parameters

Multivariate analysis revealed the effect of bacterial inoculation on plant responses to temperature stress, showing a clear separation among conditions. PCO1 explained 36.6% of the total variation, grouping plants exposed to 26 °C on the positive side and plants exposed to 36 °C on the negative side (Figure 2P). PCO2 explained 23.3% of the total variation and separated control (26 °C no inoculation) and *Pantoea* sp.-inoculated plants (both temperatures) on the negative side from plants exposed to 36 °C (no inoculation) and inoculated with *Herbaspirillum* sp. (both temperatures) on the positive side. Descriptors representing biochemical parameters superimposed on the graph were mainly correlated with higher temperature: starch, APx, sugars, proteins, CAT, PC, and ETS with *Pantoea* sp. inoculation; proline and chlorophyll b with *Herbaspirillum* sp. inoculation; and GSTs, LPO, and chlorophyll a with no inoculation.

### 2.4. Lipids

Considering the two strains, only *Pantoea* sp. was able to decrease lipid peroxidation of maize plants at both temperatures (statistically significant at 36 °C with a reduction of 65%). To understand the alterations underlying the role of *Pantoea* sp. in membrane protection, we analyzed the lipid profile of plants inoculated with this strain and compared it with that of non-inoculated plants at both temperatures.

#### 2.4.1. Multivariate Analysis of Lipids

Lipidomics analysis was carried out on *Zea mays* leaf tissue grown at 26 (C) and 36 (T) °C with and without *Pantoea* sp. (P) inoculation, resulting in significant differences in 191 lipids: 11 fatty acids, 26 glycerolipids, 42 galactolipids, 79 sphingolipids, and 33 other lipids. Based on these changes, it was possible to carry out a multivariate analysis that demonstrated the impact of temperature and inoculation on the lipid profile of *Zea mays* leaves.

**Figure 3:**
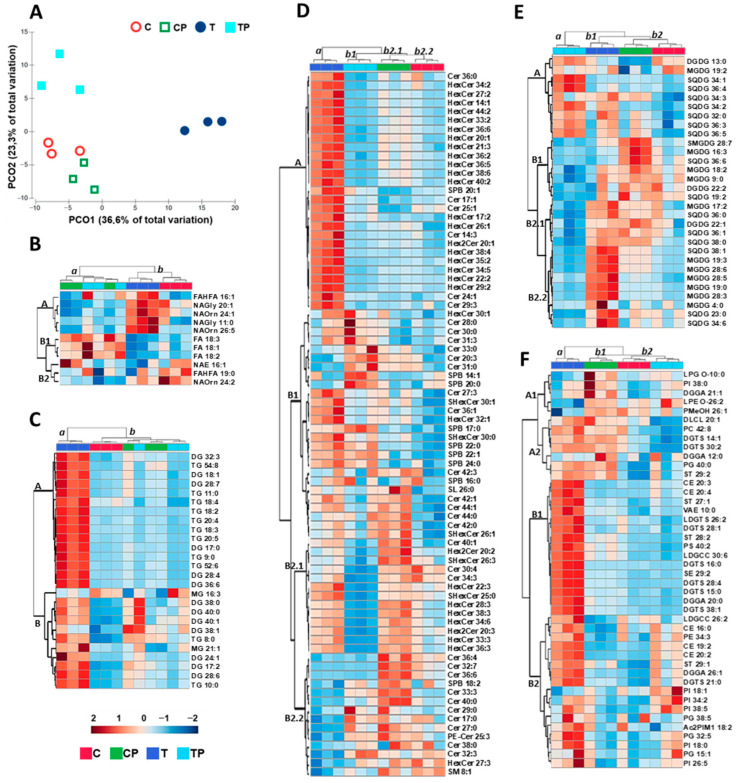
Lipid profile of *Zea mays* leaves grown under different conditions: 26 °C without *Pantoea* sp. (C); 26 °C with *Pantoea* sp. (CP); 36 °C without *Pantoea* sp. (T); and 36 °C with *Pantoea* sp. (TP). (**A**) Principal Coordinates Ordination of MCR-ALS resolved components’ peak area. Hierarchical clustering heatmaps of the identified lipids that presented significant concentration changes in *Zea mays* leaves growing in the same conditions: (**B**) fatty acids (FA—fatty acid, FAHFA—fatty acyl esters of hydroxy fatty acid, NAE—N-acyl ethanolamine, NAGly—N-acyl glycine, NAOrn—N-acyl ornithine); (**C**) glycerolipids (MG—monoacylglycerol, DG—diacylglucerol, TG—triacylglycerol); (**D**) sphingolipids (Cer—ceramid,; Hex2Cer—dihexosylceramide, HexCer—hexosylceramide, SHexCer—sulfated hexosyl ceramide, PE-Cer—phosphatidylethanolamine-Cer, SPB—sphingoid base, SL—sulfonolipid, SM—sphingomyelin); (**E**) galactolipids (MGDG—monogalactosyldiacylglycerol, SMGDG—semino monogalactosyldiacylglycerol, DGDG—digalactosyldiacylglycerol, SQDG—sulfoquinovosyl diacylglycerol); and (**F**) glycerophospholipids and other lipids (CE—cholesteryl ester, ST—sterol lipid, SE—sterol ester, PE—phosphatidylethanolamine, PG—phosphatidylglycerol, PI—phosphatidylinositol, Ac2PIM1—diacylated form of the phosphatidylinositol mono-mannoside PIM1, LPE—lyso phosphatidylethanolamine, LPG—lyso phosphatidylglycerol, PC—phosphatidylcholine, DLCL—dilyso-cardiolipin, DGGA—Diacylglyceryl-A-D-Glucuronide, DGTS—diacylglyceryl trimethyl homo-Ser, LDGCC—lyso diacylglyceryl-carboxyhydroxy-methylcholine, LDGTS—lyso diacylglyceryl trimethylhomo serine, PMeOH—phosphatidylmethanol, PS—phosphatidylserine, VAE—vitamin A fatty acid ester). Lipid clusters are represented with uppercase letters, and condition clusters with lowercase letters.

PCO1 (Figure 3A) explained 65% of the total variation and separated non-inoculated plants grown at 36 °C (T) on the positive side from the other three conditions (C, CP, and TP) on the negative side of Axis 1. Heat stress (T) imposed marked alterations in the lipid profile of *Zea mays* leaves when compared to the control condition (C), but inoculation (TP) reduced the impact of temperature on the lipid profile. PCO2 underlined the separation between TP on the positive side and C and CP on the negative side of Axis 2. The two conditions at 26 °C (C, CP) were positioned in the same quadrant, showing that inoculation at the control temperature did not cause notable differences in the lipid profile of the leaves.

Lipid family analysis allowed for detailed identification of the alterations induced by the tested conditions.

#### 2.4.2. Fatty Acids

Heatmap analysis (Figure 3B) showed two distinct groups: group a formed by inoculated conditions (CP and TP) and group b formed by non-inoculated conditions (C and T). Both CP and TP showed decreases in the fatty acids (FAs) from group A and increases in most of the FAs from group B, with no clear difference between the two conditions. The effect of temperature was more pronounced in non-inoculated plants, with a clear separation between the C and T conditions. T showed higher increases in FAs from group A, decreases in FAs from groups B and C, and increases in FAs from group B2. Group A was mostly composed of fatty acid–amino acid conjugates, which were 75% saturated or with a low degree of unsaturation. The B1 group contained 18-carbon unsaturated fatty acids. Group B2 contained fatty acyl esters.

#### 2.4.3. Glycerolipids

The glycerolipid heatmap (Figure 3C) separates heat-stressed and non-inoculated plants (T) from other conditions (C, CP, and TP), with inoculation being dominant over the temperature effect because CP and TP are more related. Glycerolipid composition was divided into two main groups (A and B). Group A included most (83%) triacylglycerols (TGs) and diacylglycerols (DGs) with a higher degree of unsaturation. These lipids increased under T conditions and decreased or did not change under the C, CP, and TP conditions. Group B included mono-, di-, and triacylglycerols, most of which were saturated (36%) or with a low degree of unsaturation (45%), which also increased under T conditions. All showed a steady decrease under the C condition, and some also increased under the CP and TP conditions. MG 16:3 and DG 38:1 differed from the general trend to increase in the T condition. MG 16:3 increased in the C condition, and DG 38:1 increased in the CP and TP conditions.

#### 2.4.4. Sphingolipids

The sphingolipid heatmap (Figure 3D) also separates T from the other three conditions, and the samples at 26 °C (C and CP) are more similar to each other than to TP. Twenty-seven sphingolipids, mainly polyunsaturated hexosylceramides (HexCer), were higher in T than in the other conditions (group A). Twenty-six sphingolipids, mainly saturated or monounsaturated ceramides (Cers) and sphingoid bases (SPBs), were more abundant at 36 °C (T and TP) than at 26 °C (C and CP) (group B1). The other sphingolipids from group B2.1 were mainly long-chain highly unsaturated HexCer, which was more abundant in CP and less abundant in TP, and from group B2.2 were mainly long-chain saturated Cer, which was less abundant in T.

#### 2.4.5. Galactolipids

The galactolipid profile (Figure 3E) highlighted three groups formed by TP, T, and C conditions. Lipids were grouped into four groups (A, B1, B2.1, and B2.2), with group A mainly formed by long-chain SQDG (higher than 32C), most of which was unsaturated. The levels of these galactolipids increased at 36 °C, particularly under the TP condition. B1 included monogalactosyldiacylglycerol (MGDG), digalactosyldiacylglycerol (DGDG), sulfoquinovosyl diacylglycerol (SQDG), and semino monogalactosyldiacylglycerol (SMGDG), mostly with two or more double bonds. *Pantoea* sp. increased the concentration of B1 lipids at 26 °C and decreased the concentration at 36 °C. Group B2.1 contained different galactolipids (SQDG, MGDG, and DGDG), in general being unsaturated or monounsaturated and increasing in T and decreasing in TP conditions. Galactolipids from B2.2 were mainly unsaturated MGDG, and there was evidence of a higher concentration in T compared to the remaining conditions.

#### 2.4.6. Glycerophospholipids and Other Lipids

Analysis of glycerophospholipids and other lipids (Figure 3F) also separated the T condition (group a) from the three other conditions (group b). Plant inoculation at 36 °C (TP) brought the lipid profile closer to the control condition (C) (group b2). Four main lipid groups (A1, A2, B1, and B2) were identified. Group A1 included LPG, PI, DGGA, LPE, and PMeOH, mostly saturated or with low degrees of unsaturation, which decreased in the T condition and increased in control-inoculated plants (CP). Group A2 included different lipids (DLCL, PC, DGTS, DGGA, PG, and ST), mostly saturated or with low degrees of unsaturation, which decreased under T and CP conditions. Group B1 was a heterogeneous group (CE, ST, VAE, LDGTS, DGTS, ST, PS, LDGCC, DGTS, SE, and DGGA) with different numbers of double bonds (0–6) and chain lengths (10–40 carbon atoms) that had higher concentrations under the T condition. Group B2 included different lipids (LDGCC, CE, PE, ST, DGGA, DGTS, PI, Ac2PIM1, and PG) with different numbers of double bonds (0–5) and chain lengths (15–38 carbons) at lower concentrations at 26 °C. Temperature (T) increased B2 lipid concentrations, and inoculation at 36 °C (TP) mitigated some of the increases. PI 18:1 was an exception in this group as it did not increase with temperature.

## 3. Discussion

Climate change scenarios predict temperature increases as a major consequence, with severe impacts on plant growth due to cell damage induced by the generation and accumulation of reactive oxygen species, protein misfolding, and disruption of the photosynthetic machinery [19]. To clarify the effects of rhizobacterial inoculation on plant growth and heat stress tolerance, morphometric, biochemical, and lipidomic approaches were used to compare the responses of maize plants grown under different temperatures and inoculation conditions.

### 3.1. Plant Emergence and Growth

The results of our study evidenced that an increase of 10 °C did not significantly change shoot and root dry weight in non-inoculated plants, but did change plant emergence.

Under field conditions, heat stress is often accompanied by drought stress, and most maize studies have considered the combined effects of heat and water stress. Therefore, it is important to evaluate the isolated effect of temperature as a factor conditioning crop growth to unveil the plant processes that are most sensitive to temperature. The results of our study, which only considered heat stress, showed that an increase of 10 °C did not significantly change shoot and root dry weights in non-inoculated plants, evidencing the relief conferred by water availability to plants under heat stress. Plant tolerance to high temperatures may also be influenced by interactions with microorganisms. The strains used in our study increased the dry weight of the inoculated plants under control and heat stress conditions. Breedt et al. [20] found an increased yield ranging from 24 to 34% in temperature-stressed maize plants inoculated with *Paenibacillus alvei*, *Bacillus safensis*, *Bacillus pumilus*, and *Brevundimonas vesicularis*, and Notununu et al. [21] reported an increase of 28.7 and 47.9% in the growth of maize exposed to the same abiotic stress when inoculated with *Lelliottia amnigena* and *Leclercia adecarboxylata*, respectively.

Temperature is an important factor for seed germination and emergence. Moderate temperatures of approximately 26–29 °C are required for the adequate germination of maize seeds. Above or below this temperature range, germination is affected, and maize does not germinate when the temperature is too high (>45 °C) or too low (<6.2 °C) [22]. Our study showed that seed emergence was lower at 36 °C than at 26 °C. Several studies have reported the effects of bacterial inoculation on maize seed germination. Dos Santos et al. [23] reported an increase in seed germination rate and early development in maize inoculated with strains of *Burkholderia cepacian* and *Burkholderia graminis*. Houida et al. [24] found that different bacterial strains significantly increased maize germination rate (26–78%), root elongation (67–84%), and seedling fresh and dry weights; *Aeromonas encheleia* was the strain with greater influence on seed germination due to its strong ability to produce indole-3-acetic acid (IAA), and along with *Pseudomonas azotoformans*, was the strain most proficient at enhancing seedling root elongation and biomass. Both the *Pantoea* sp. and *Herbaspirillum* sp. strains used in this study are able to produce IAA at high temperatures (unpublished data), explaining the increase in germination rate. Moreover, Weisskopf et al. [25] and Rani et al. [26] reported that bacterial volatile organic compounds can trigger seed germination. The two strains used in our study also have the ability to produce VOCs and increase plant growth under heat stress (unpublished), which could be another mechanism that explains the germination rate increase under inoculated conditions observed at 36 °C. These findings underline the importance of bacterial inoculation during the early plant development stages, especially when germination occurs under heat stress.

### 3.2. Biochemical Response

#### 3.2.1. Heat Stress Effect

Different studies have reported that high temperatures negatively affect chloroplast membranes and chlorophyll molecules, thereby reducing the photosynthetic capacity [27]. During heat stress, chlorophyll b was shown to have protective effects on the photosynthetic apparatus [28]. High temperature induced a slight increase in chlorophyll a in non-inoculated plants, but chlorophyll b was significantly increased. These results suggest a compensatory mechanism for the described decrease in photosynthetic capacity when the temperature increases [29]. Heat-stressed plants have also been reported to have increased proline levels because of the ability of this amino acid to scavenge reactive oxygen species, stabilize proteins and cell structures, and regulate cell metabolism and genetic expression [30,31]. In our study, proline was significantly increased by temperature stress, indicating a protective role of proline in temperature-stressed maize plants.

The activity of ETS in the mitochondria provides information on plant metabolic activity and respiratory potential, with a study describing a decrease in ETS activity in abiotically stressed plants [32]. However, the stress response is energy-demanding, and increases in ATP synthesis by oxidative phosphorylation have been reported [33]. In our study, ETS was increased by temperature stress, providing higher levels of ATP and the reducing power necessary for metabolic pathways that restrain temperature stress effects, such as excessive ROS scavenging.

Our results also showed that soluble protein levels increased in heat-stressed plants. A high proportion of soluble proteins are enzymes whose activity adjusts cell metabolism and plays a crucial role in the plant stress response [34].

The activity of ROS-scavenging enzymes, such as CAT, SOD, GSTs, GPx, and APx, is well documented and crucial for reducing oxidative damage generated by temperature [35]. Our results indicated an increase in the activity of all antioxidant enzymes studied, confirming temperature as a pro-oxidant factor. The increase in SOD and APx activity, enzymes that can be found in chloroplasts [36], may indicate the excess of ROS produced in the photosynthetic electron transport chain at high temperature.

Lipid peroxidation, a marker of membrane oxidative damage caused by ROS, increases in tissues when plants are exposed to temperature stress [37]. Protein carbonylation, a marker of oxidative stress in proteins, is also increased in heat-stressed plants [38]. Despite the increase in the antioxidant system, our results showed that oxidative stress imposed by high temperature was not completely neutralized, since cell damage both in membranes (lipid peroxidation) and soluble proteins (protein carbonylation) was higher in plants exposed to temperature stress than in the control.

#### 3.2.2. Inoculation Effects at Control Temperature

Several studies have described the positive effects of inoculation on a variety of crops, such as wheat [39], maize [40], tomato [41], and beans [42]. However, there is a lack of studies addressing the biochemical responses behind these effects. Under the control temperature (26 °C), our study demonstrated that *Herbaspirillum* sp. and *Pantoea* sp. were able to increase protein content and ETS activity in maize plants when compared to non-inoculated plants, as reported for other systems [43]. Moreover, *Herbaspirillum* sp. inoculation significantly increased photosynthetic pigments, whereas *Pantoea* sp. did not have a significant effect, indicating a differential response depending on PGPB. Rojas et al. [44] and Ren et al. [45] reported that PGPB inoculation increased chlorophyll content.

According to our results, bacterial inoculation reduced the cellular oxidative level, with lower lipid peroxidation in inoculated plants than in non-inoculated plants. Some beneficial microorganisms can enhance the activity of antioxidant enzymes in plants [46]. In our study, *Herbaspirillum* sp. was able to significantly increase GST, SOD, and CAT activities, and *Pantoea* sp. significantly induced the activities of GSTs, SOD, CAT, and APx at the control temperature. This increase in antioxidant protection may lead to less damage over time, increasing the lifespan of components and molecules susceptible to oxidation and reducing the metabolic effort to replace them, leaving more energy and reducing power available for other cellular processes, such as growth.

#### 3.2.3. Inoculation Effect Under Heat Stress

The increase in soluble protein and metabolism demands higher energy (provided by ETS activity). Our results demonstrated that both strains had significantly increased protein content and ETS activity at 36 °C.

According to our study, *Herbaspirillum* sp. significantly increased GST and CAT activities, and *Pantoea* sp. significantly increased CAT and APx activities in heat-stressed plants compared to heat-stressed non-inoculated ones. The main difference between the two strains was the higher APx activity (77%) in *Pantoea* sp. than in *Herbaspirillum* sp. An increase in antioxidant enzymes may explain the significant reduction in membrane damage (lower LPO levels), especially in *Pantoea* sp.-inoculated plants. In other studies, *Bacillus safensis*, *Ochrobactrium pseudogrignonense*, and *Bacillus cereus* were reported to activate antioxidant signaling in plants under heat stress, leading to enhanced redox enzyme activity [47]. The protective effect on membranes conferred by *Pantoea* sp. may be linked to the increase in APx activity. APx has been reported to be a key determinant of plant stress tolerance, decreasing ROS levels, especially in chloroplasts [36]. Thus, our results demonstrated the protective effect PGPB inoculation may have on plant membranes. Moreover, chlorophyll a and b levels decreased in heat-stressed plants inoculated with *Pantoea* sp. to values similar to non-stressed plants.

Temperature induces changes in the plant lipid profile [10], and bacteria can modulate the lipid composition of plant membranes [48], making them more stable and less susceptible to thermal stress. Since *Pantoea* sp. induced lower LPO levels at the higher temperature, it can be relevant to determine the effect this strain can have on the plant lipid profile, rendering membranes less susceptible to lipid peroxidation. This approach may bring novel information to understand the effects that PGPB may have on factors inducing oxidative stress and on plant heat tolerance.

### 3.3. Lipid Profiles

Multivariate analysis carried out on lipid data from *Zea mays* grown at different temperatures and inoculation conditions allowed us to recognize the impact of these two variables on the leaf lipidome. A clear separation between the lipid profiles of the control (26 °C) and stress temperature (36 °C) was noticeable. These results are in accordance with the literature that describes lipid profile alterations induced by temperature increases in different crops and model species [49,50]. Under high temperatures, phospholipid fatty acid tails become less rigid and reach sufficient kinetic energy to overcome the intermolecular forces that hold the membrane together. This effect increases membrane fluidity and permeability and decreases membrane stability. A common heat stress effect is the higher proportion of long-chain saturated lipids in plant membranes, which allows higher membrane lipid packing and increases the number and strength of intermolecular forces between neighboring lipids, resulting in membrane stability [51]. In our study, inoculation with *Pantoea* sp. induced slight changes in the lipid profile of maize leaves at the control temperature (26 °C). However, under heat stress (36 °C), *Pantoea* sp. inoculation reversed the temperature effect and brought the lipid profile closer to the control conditions (C and CP). This effect is an important feature that ensures the impact that PGPB strain inoculation may have on plants, conferring protection against high temperatures and increasing plant tolerance [52]. Studies reporting plant lipid changes induced by PGPB inoculation are scarce [49] and further investigation is needed to elucidate novel lipid functions and profiles [17].

In plants, fatty acids are components of glycerol-containing lipids, sphingolipids, and extracellular lipids (cuticular waxes and lipid polyesters). The predominant unsaturated fatty acids (UFAs) in plants are three 18-carbon (C18) lipids: oleate (18:1), linoleate (18:2), and α-linolenate (18:3) [53]. These lipids play crucial roles and are strongly associated with both abiotic and biotic stresses, functioning as membrane constituents, glycerolipid modulators, and triacylglycerol carbon and energy reserves. C18 UFAs also work as intrinsic antioxidants and precursors of numerous bioactive molecules and are needed for the synthesis of extracellular barrier constituents, such as cutin and suberin [54], which are crucial for thermal insulation and protecting plants from excessive temperature [55]. Our results showed that oleate (18:1), linoleate (18:2), and α-linolenate (18:3) increased in inoculated plants at both temperatures, indicating that *Pantoea* sp. promoted changes that may have induced systemic resistance in plants, helping to cope with temperature stress (TP condition), even in the absence of stress (CP). According to Castanheira et al. [56], bacterial inoculation of ryegrass increased FA biosynthesis by 65%; however, the physiological and biochemical consequences of this change have not been addressed. In heat-tolerant rice plants, the degree of saturated fatty acids increases with temperature in both thylakoid and cellular membranes [49]. Our results are not in agreement with these studies, since neither an increase in saturated fatty acids nor a decrease in unsaturated fatty acids was observed at 36 °C. The fact that maize is a C4 plant might explain these differences. Plants with this metabolism have lower levels of oxygen in their photosynthetic tissues; therefore, thylakoid membranes are less subjected to lipid peroxidation, with less need to reduce the degree of lipid unsaturation to protect these membranes from oxidative damage. Moreover, inoculation with *Pantoea* sp. increased this effect, as the inoculated plants showed lower lipid peroxidation. The absence of hydroperoxidation of polyunsaturated lipids, such as α-linolenate, decreased hydroperoxy-octadecatrienoic acid (the hydroperoxide lipid resulting from α-linolenate), which is the precursor of jasmonic acid, a plant hormone that activates signaling transduction and programed cell death [17], thus contributing to the maintenance of metabolic homeostasis in thermostressed plants inoculated with *Pantoea* sp.

N-acyl amino acids (NAAAs) are an important class of lipid molecules in which an amino acid is linked to the acyl moiety of a long-chain fatty acid. These lipids are amphiphilic molecules that allow interactions with different molecules, modifying membrane properties and influencing membrane fluidity and integrity [57]. Most fatty acids that increased with temperature in our study were lipid–amino acid conjugates, 75% of which were saturated or with a low degree of unsaturation, evidencing a well-known mechanism of plants to maintain membrane stability under increased temperature [58], which decreases the susceptibility of lipids to hydroperoxidation (unsaturated lipids) [17]. Amino fatty acids have been reported to help plants cope with environmental stresses such as drought, salinity, and extreme temperatures [59]. Some amino fatty acids are involved in the regulation of phospholipid metabolism, thereby influencing their synthesis and membrane turnover. Moreover, amino fatty acids are also involved in signaling pathways that regulate stress-responsive genes, interacting with plant hormones such as abscisic acid and jasmonic acid, which are involved in stress responses, including temperature stress [60]. The synthesis of secondary metabolites, including phenolic compounds and flavonoids, which can be involved in the plant response to temperature stress, is also affected by amino fatty acids [61]. In the present study, there was a clear increase in NAAAs in plants subjected to higher temperatures, confirming the importance of these lipids in plant heat stress tolerance. Interestingly, *Pantoea* sp. reduced NAAAs, still allowing inoculated plants to better cope with temperature increase, which may be linked to lower peroxidation levels.

In our study, temperature-increased glycerolipids indicate lipid membrane remodeling, with increased accumulation of DG and TG, indicating that fatty acids from structural lipids are redirected in the transient assembly of TG [62]. DGs are precursors of glycolipids, storage lipids, and the major structural phospholipids, which together account for approximately 90% of all plant lipids. In plants DG content is relatively low since DG molecules are rapidly phosphorylated to phosphatidic acid (PA). PAs are key lipid signaling molecules, and their involvement in plant stress response, metabolism, and development has been reviewed [63]. Heat stress also leads to TG accumulation in plant tissues and has been consistently reported in different species [64]. In *Arabidopsis thaliana* leaves exposed to 37 °C, TG accumulated rapidly in the first 3 h and reached a steady-state level after 6 h [65]. Compared to other abiotic stresses, such as cold, salt, drought, and high light intensity, heat was the strongest inducer of TG accumulation after short-term stress exposure [66]. In the same study, the level of total fatty acids remained unchanged during heat stress, indicating that heat-induced TG accumulation was not driven by massive de novo fatty acid synthesis but rather by significant lipid remodeling [66]. Our results on glycerolipid analysis separated the high-temperature non-inoculated condition (T) from other conditions (C, CP, and TP), supporting the effect of high temperature on glycerolipids described in other studies. Changes triggered by heat stress (T) were reverted to control (non-inoculated plants at 26 °C) levels by bacteria (TP), highlighting the role of *Pantoea* sp. inoculation in the *Zea mays* response to temperature. An overview of our MG results evidenced that MG 16:3 tended to decrease, and MG 21:1 increased with temperature. In fact, the literature points to an increase in longer-chain lipids with lower degrees of unsaturation as a mechanism to maintain membrane stability at higher temperatures [11], evidencing once more that plant membranes can be protected from the deleterious effects of oxidative stress generated by high temperatures decreasing unsaturated fatty acids, which are more prone to peroxidation [67]. Chloroplast ultrastructure was also reported to change in response to temperature, with *A. thaliana* chloroplasts being reported to swell and the number and size of internal lipid droplets increasing under heat stress [68]. The accumulation of TGs, able to sequester the released cytotoxic free fatty acids and formation of lipid droplets [69], could prevent these toxic FAs from interacting with chloroplast and cytosolic molecules and reduce the negative impact of peroxidation. Lipid droplet formation mechanisms could explain the increase in DGs and TGs in our study under temperature stress.

Photosynthesis is highly dependent on the stability of the chloroplast membrane lipid moiety in which protein complexes are embedded. The main thylakoid membrane lipids are the galactolipids MGDG, DGDG, and SQDG, and the phospholipid PG [70]. MGDG and DGDG play essential roles in chloroplast development and maintenance of the electron transport system [70], fatty acid synthesis [71], and cytochrome complex photoreduction [72]. They may also be involved in carbohydrate transport [73]. DGDG concentration was also related to chlorophyll content and photosynthesis levels [74]. According to our study, temperature led to an increase in MGDG and DGDG. Under temperature stress, highly unsaturated chloroplast galactolipid chains are prone to oxidation [75], causing membrane instability and disruption of photosynthesis. The increased saturation of lipids under abiotic stress is a part of membrane lipid remodeling and has been shown to stabilize membrane fluidity and photosynthesis [76]. In our study, galactolipid concentrations increased under heat stress, maintaining the degree of unsaturation and increasing chain length. Thus, the membrane’s vulnerability to ROS did not decrease (the degree of unsaturation did not change); however, the membrane stability increased (higher chain length). One of the mechanisms reported to protect thylakoid membranes from oxidative stress is a group of lipid-based molecules, the tocopherols, which are powerful antioxidants that play an important role in restricting lipid peroxidation [77] and preserving the photosynthetic apparatus. Figueira et al. [78,79] observed increases in α-tocopherols in *Juncus maritimus* and *Halimione portulacoides* exposed to mercury stress. Spicher et al. [80] also reported increases in tocopherols in *Solanum lycopersicum* grown at high temperature. In our study, tocopherols did not change significantly among the conditions tested, indicating that thylakoid membranes were vulnerable to ROS oxidative attack with an impact on their functionality under heat stress, or demonstrating that membrane lipid integrity was preserved by the action of other antioxidant mechanisms (molecular or enzymatic). In fact, our study described the increase in APx, an antioxidant enzyme present in chloroplasts that efficiently scavenges ROS, in *Pantoea* sp.-inoculated plants exposed to 36 °C. The conical shape of MGDG, the cylindrical shape of the DGDG headgroup, and the MGDG/DGDG ratio are related to grana stacking and photosynthetic functioning [52]. MGDG may stabilize membrane regions of concave curvature, and a high proportion of MGDG in chloroplast membranes has been suggested to be important for driving the formation of thylakoid stacks [69]. Our results showed an increase in the MGDG/DGDG ratio under increased temperature, indicative of membrane stabilization and heat stress response. High temperatures induced SQDG accumulation in drought-resistant wheat leaves and chloroplasts [81]. Data presented in the literature reported that temperatures of 30–43 °C induced accumulation of SQDG with a high content of palmitic and oleic acid residues in *Atriplex lentiformis* plants [82]. In our study, temperature also increased the SGDG concentration in *Zea mays*, confirming its relevance in stress response.

Sphingolipids constitute up to 10% of higher plants’ lipids [83]. Plant sphingolipids perform important structural functions that maintain membrane integrity. An important feature is their clustering with sterols, forming highly dynamic microdomains called lipid rafts located in the plasma membrane [84]. These rafts have previously been shown to be enriched in membrane zones with higher concentrations of proteins that perform various functions in response to biotic and abiotic stresses [85], thus being important for the maintenance of membrane functioning.

Sphingolipids are also involved in different processes such as regulatory/signaling pathways, cell-to-cell interactions [84], abscisic acid (ABA)-dependent stomatal closure [86], programmed cell death [87], membrane stability [88], drought and salt tolerance [89], pollen development [90], cell division and growth, and plant–microbe interactions [91]. In this study, 80 sphingolipids (HexCer, Cer, and SPB were the most abundant) changed significantly among the conditions, and the separation between T and the other three conditions (TP, CP, and C) was clear. Cheong et al. [92] reported decreases in sphingolipids (Cer and HexCer) in different wheat varieties exposed to cold stress. Krawkczyk et al. [93], reported that heat stress led to an additional increase in all sphingolipid subclasses, especially the levels of SPB phosphates and HexCer in tobacco. According to our results, higher temperature increased sphingolipid concentration, which may indicate a higher presence of membrane microdomains promoting membrane stability and membrane-associated protein functionality.

Glycerophospholipids and other lipids also showed significant changes among conditions. The general trend was an increase with temperature (T) and the presence of *Pantoea* sp. (TP) decreased the concentrations to control levels (C). Glycerolipids that contain phosphate groups in their structure are called glycerophospholipids and they are dominant in cell membranes, providing stability, fluidity, and permeability [17,94]. Moreover, they are required for the proper functioning of membrane proteins, receptors, and ion channels, and act as reservoirs for second messengers and their precursors [95]. Important plant phospholipids include PA, PC, PE, PI, PS, PG, and their respective lysophospholipids such as lysophosphatidic acid (LPA) and lysophosphatidylcholine (LPC). The major membrane phospholipids of plant cells are the bilayer PC and the non-bilayer PE lipids, as well as PI and PS [69]. Common features of phospholipids are their non-polar fatty acyl chains and polar head groups, which interact with the water-soluble environment. Phospholipids play an important role in regulating cell activities, such as membrane rearrangement, cytoskeletal dynamics, phosphorus deficiency response, and cold stress response [96]. Several studies reported that phosphoinositide, a cellular signaling molecule produced from PI, increases in response to drought and cold stress [89,97]. Narayanan et al. [95] also found that the PC and PE contents generally decreased in wheat under high-temperature stress. Li et al. [98] found that heat stress increased PC content with no change in PE levels in leaves of *Atriplex lentiformis*. According to our results, the PI, PC, and PE increased with temperature. Chen et al. [68] reported an increase in PA levels with temperature in *Zea mays*. PA levels can change rapidly, serving as a signaling molecule that regulates a range of different physiological processes, such as activities of kinases, phosphatases, phospholipases, and proteins involved in membrane trafficking, Ca^2+^ signaling, and oxidative burst [99], and modulating plant responses to different abiotic and biotic stresses [100]. In our study, no significant changes were observed in the PA levels. PA is involved in the rapid response to stress and is then converted into DG [101]. In our study, plant sampling was performed approximately one month after the onset of heat stress, and the increase in PA may have no longer been detectable.

Plant sterols are essential constituents of the cell membrane and mitochondrial outer membrane that regulate membrane fluidity and permeability by restricting the mobility of fatty acyl chains. In membranes, plant sterols are associated with glycosphingolipids in raft domains, which determine the membrane location and activities of many proteins with crucial functions in plant cells. Sterols are involved in membrane temperature tolerance and in the modulation of membrane-bound enzyme activity, such as ATPases. Shiva et al. [51] reported increases in sterol levels in *A. thaliana* in response to heat stress. Similar results were obtained by Singh et al. [102] in *Withania somnifera*. According to our results, the increase in sterols with temperature may constitute a mechanism of membrane stabilization, ensuring several functions, such as transport, electrochemical potential, photosynthesis, and respiration.

Altogether, the changes observed in the different lipid families led to increased membrane stability under temperature stress. Inoculation with *Pantoea* sp. reduced the need for lipid remodeling by inducing membrane-protective mechanisms, mainly APx activity.

Table 1 provides a comparative overview of our findings along with those reported in previous studies, offering a clearer and more accessible visual representation to facilitate the reader’s understanding of the key points discussed.

## 4. Materials and Methods

### 4.1. Bacterial Strains

The bacterial strains used in this study were obtained from two agricultural soils located in Mogadouro (41.353474, −6.693088) and Vila do Conde (41.427052, −8.585773), mainland Portugal. After sampling, the soils were placed in pots with soybean plants to isolate bacteria interacting with the root system. The strains were grown for 24 h at 26 °C in an orbital shaker (160 rpm) in yeast mannitol broth (YMB) medium. Two isolates exhibited higher growth and plant growth promotion abilities (indole-3-acetic acid, siderophores, volatile organic compound production, phosphate solubilization, and antifungal activity) at 40 °C compared to the control temperature (26 °C) (unpublished). These isolates were identified at the genus level through partial 16S rRNA gene sequencing [103], and the gene sequences were submitted to GenBank. One isolate belonged to the genus *Herbaspirillum* (accession: OM986001) and the other belonged to the *Pantoea* genus (accession: OM986067).

### 4.2. Experimental Conditions

*Zea mays* seeds (Dekalb DKC 6031) were placed in 200 mL plastic pots filled with a 3:2 (*w*/*w*) peat/perlite sterile mixture under controlled day/night conditions in climate chambers (Fitoclima D1200, Aralab, Rio de Mouro, Portugal) with a 16/8 h light/dark photoperiod, 65%/60% day/night relative humidity, and 500 μmol m^2^ s^−1^ photosynthetic photon flux density (PPFD) for 32 days. Six conditions were tested: non-inoculated controls (C), inoculated with *Pantoea* sp. (P), and inoculated with *Herbaspirillum* sp. (H) at two temperature regimes: 26/21 °C day/night as the control and 36/26 °C day/night as the stress temperature. Each condition included five pots with two seeds each that were randomly distributed in the chamber. Pot inoculation was performed using 5 mL of fresh culture solution of *Herbaspirillum* sp. or *Pantoea* sp. (8 × 10^8^ cells/mL) grown in YMB for 24 h at 26 °C in an orbital shaker (160 rpm). Germination rate was assessed as the number of plantlets that emerged after 5 days. Plants were fertilized on days 4 and 15 with 10 mL N:P:K 5:8:10 (half-strength solution Complesal, Bayer CropScience). The plants were watered twice daily up to 80% field capacity (FC). The pot weight was monitored, and the percentage of FC was maintained by adding the amount of water lost.

At the end of the experiment, plants were collected and shoots and roots were separated, washed first in tap water, and then washed in deionized water to remove substrate particles. Shoot and root length and fresh and dry (dried at 60 °C until constant weight was attained) weights were determined. Leaf samples were collected for immediate determination of photosynthetic pigments. Leaf samples were also stored (−80 °C) for further analysis.

After harvesting, the substrate was homogenized and 1 g of each pot was collected and added to a tube containing 9 mL of deionized water (10^−1^ dilution) to determine the colony-forming units (CFUs). Serial dilutions (10^−2^, 10^−3^, 10^−4^, 10^−5^, and 10^−6^) were prepared, and 1 mL of each dilution was plated in yeast mannitol agar (YMA) medium in triplicate and incubated for 3 days at 26 °C. Colonies were counted and the results were expressed as colony-forming units per gram of soil (CFU/g soil).

### 4.3. Photosynthetic Pigments

Fresh samples were milled in liquid nitrogen, followed by pestle and mortar homogenization in 80% acetone (1:2 *w*/*v*), and allowed to rest for 45 min in the dark at 4 °C. Extracts were then centrifuged at 4000× *g* for 5 min, and the pigment content was determined following the method described by Wellburn and Lichtenthaler [104]. Absorbance was measured at 663, 646, and 470 nm, and chlorophyll a and b and carotenoids were determined using the equations proposed by Wellburn and Lichtenthaler [104]. Results were expressed in micrograms per gram of fresh weight (µg/g FW). The remaining plant material was milled in liquid nitrogen and stored at −80 °C.

### 4.4. Soluble and Insoluble Carbohydrates

Frozen samples were homogenized in deionized water using a mortar and pestle. Extracts were centrifuged at 10,000× *g* for 4 min. The supernatant was collected and used to determine soluble carbohydrate content. The pellet was resuspended in 2.5 mM sulfuric acid (1:2 *w*/*v*) and incubated at 95 °C for 1 h to hydrolyze the insoluble storage carbohydrates (starch). Quantification of both soluble and insoluble carbohydrates was performed using the method described by Dubois et al. [105] with some modifications. To 15 µL of sample, 900 µL 98% sulfuric acid and 150 µL 5% phenol were added. The mixture was then incubated for 2 h at room temperature. Samples were then centrifuged at 10,000× *g* for 5 min, the supernatant was collected, and the absorbance was measured at 492 nm. Glucose standards (1–10 mg/mL) were used. Results were expressed in milligrams of glucose per gram of fresh weight (mg/g FW).

### 4.5. Proline

Frozen samples were homogenized using a mortar and pestle in 3% sulfosalicylic acid (1:2 *w*/*v*) and centrifuged at 12,000× *g* for 10 min at 4 °C. Supernatants were collected and used for determination of proline, following the method described by Bates et al. [106] with some modifications. Acid ninhydrin (250 µL) and glacial acetic acid (250 µL) were added. After incubation for 1 h at 100 °C, the reaction was stopped by placing samples on ice. Absorbance was measured at 520 nm and proline (Sigma-Aldrich, St. Louis, MO, USA) standards (0–1 mg/mL) were used. Results were expressed in micrograms of proline per gram of fresh weight (μg/g FW).

### 4.6. Lipid Peroxidation

Frozen samples were homogenized using a pestle and mortar in 20% (*v*/*v*) trichloroacetic acid (1:2 *w*/*v*) and centrifuged at 12,000× *g* for 10 min. The supernatant was collected and used for quantification of thiobarbituric acid reactive substances (TBARSs) according to the methodology described by Buege and Aust [107]. Absorbance was measured at 532 nm, and TBARSs were estimated using the molar extinction coefficient for malondialdehyde (MDA) (1.56 × 10^5^ M^−1^ cm^−1^). Results were expressed in nmol of MDA equivalents per gram of fresh weight (nmol/g FW).

### 4.7. Protein, Protein Carbonylation, Electron Transport System, and Antioxidant Enzymes

Frozen samples were homogenized using a pestle and mortar in sodium phosphate buffer (50 mM sodium dihydrogen phosphate monohydrate, 50 mM disodium hydrogen phosphate dihydrate, 1 mM ethylenediaminetetraacetic acid disodium salt dihydrate (EDTA), 1% (*v*/*v*) Triton X-100, 1% (*v*/*v*) polyvinylpyrrolidone (PVP), 1 mM dithiothreitol (DTT) (pH 7.0) (1:2 *w*/*v*), and centrifuged at 3300× *g* for 5 min at 4 °C (electron transport system activity) or 12,000× *g* for 10 min at 4 °C (other biochemical parameters). The supernatants were collected and used immediately or stored at −80 °C for determination of protein content, protein carbonylation (PC), electron transport system (ETS) activity, superoxide dismutase (SOD), catalase (CAT), glutathione S-transferase (GST), glutathione peroxidase (GPx), and ascorbate peroxidase (APx) activity.

Protein content was determined using the method described by Robinson and Hodgen [108]. To 50 µL of sample, 250 µL of biuret reaction solution was added. Samples were then incubated in the dark for 10 min at room temperature. Absorbance was measured at 540 nm, and bovine serum albumin (BSA) (Sigma-Aldrich, St. Louis, MO, USA) was used as the standard (5–40 mg BSA/mL). Results were expressed in mg protein per gram of fresh weight (mg/g FW).

Protein carbonylation (PC) was assessed by quantifying carbonyl groups (CGs) using the 2,4-Dinitrophenylhydrazine (DNPH) alkaline method described by Mesquita et al. [109] with modifications described by Udenigwe et al. [110]. In a 96-well microplate, 120 µL of sample or blank (extraction buffer) was added, followed by 120 µL of DNPH. After 10 min of incubation at room temperature, 60 µL of sodium hydroxide (NaOH 6M) was added, and the reaction was incubated for 10 min. Absorbance was read at 450 nm and calculated using the molar extinction coefficient of CG (22,308 mM^−1^ cm^−1^). Results were expressed in micromoles of CG per gram of fresh weight (µmol/g FW).

Electron transport system (ETS) activity was measured using the King and Packard method [111]. To 37.5 µL of sample, 107 µL of balanced salt solution (BSS) buffer (0.13 M Tris-HCl, 0.3% (*v*/*v*) Triton X-100, pH 8.5), 35.7 µL of reduced nicotinamide adenine dinucleotide phosphate NAD(P)H (1.7 mM NADH and 250 µM NADPH), and 71.4 µL of 8 mM *p*-iodonitrotetrazolium (INT) were added; the reaction started with the addition of INT. The absorbance was read at 490 nm for 10 min at intervals of 25 s. The amount of formazan formed was calculated using the molar extinction coefficient of formazan (15,900 M^−1^ cm^−1^), and the results were expressed in micromoles of formazan formed per min per gram of fresh weight (μmol/min/g FW).

Superoxide dismutase (SOD) activity was determined by the conversion of nitro blue tetrazolium (NBT) by the superoxide-free radicals into NBT diformazan using the methodology described by Beauchamp and Fridovich [112]. To 25 µL of sample, 25 µL of xanthine oxidase and 250 µL of reaction buffer with NBT were added and incubated for 20 min at room temperature with orbital rotation. Absorbance was measured at 640 nm. One unit of enzyme activity (U) represented a 50% reduction in NBT. Results were expressed in units of enzyme (U) per gram of fresh weight (U/g FW).

Catalase (CAT) activity was determined using the methodology described by Johansson and Borg [113]. In a 96-well microplate, 25 µL of the sample was added, followed by 125 µL of reaction buffer (1M K_2_HPO_4_ and 1M KH_2_PO_4_, pH 7.0), 37.5 µL of methanol, and 25 µL of 35.28 mM hydrogen peroxide (H_2_O_2_). After 20 min incubation at room temperature, 37.5 µL of 10 M potassium hydroxide (KOH) and 37.5 µL of 34.2 mM Purpald were added, followed by 10 min incubation at room temperature, and then 12.5 µL of potassium periodate (KIO_4_) was added. After 5 min incubation, the absorbance was read at 540 nm. Catalase activity was determined using a standard curve of formaldehyde (5–150 µM), and results were expressed in milliunits of enzyme (mU) per gram of fresh weight (mU/g FW).

The activity of glutathione S-transferase (GST) was determined according to Habig et al. [114]. The reaction buffer (100 mM potassium phosphate buffer, pH 6.5, 10 mM reduced glutathione, and 60 mM 1-chloro-2,4-dinitrobenzene) was added to the sample in a 2:1 (*v*/*v*) ratio. The reaction was followed for 20 min at 15 s intervals at 340 nm. The activity of GST was determined using the extinction coefficient of the glutathionyl-dinitrobenzene (GS-DNB) conjugates (9.6 mM^−1^ cm^−1^). GST activity was expressed in milliunits of enzyme (mU) per gram of fresh weight (mU/g FW).

GPx activity was determined using the Paglia and Valentine method [115]. The determination of GPx activity was based on the ability of the enzyme to reduce cumene hydroperoxide by the oxidation of GSH to GSSG. The GSSG formed could be reduced again to GSH by the activity of glutathione reductase, consuming NADPH in proportion to the amount of GSSG reduced, and thus to GPx activity. The absorbance was measured at 340 nm. The amount of NADPH consumed was calculated using the micromolar extinction coefficient of 0.00622 μM^−1^ cm^−1^ for NADPH and expressed in milliunits of enzyme activity per gram of fresh tissue (mU/g FW).

Ascorbate peroxidase (APx) activity was measured by following the method adapted from Nakano and Asada [116]. The reaction buffer (185 µL/well), consisting of 50 mM potassium phosphate buffer (pH 7.0) and 0.25 mM ascorbic acid, was dispensed into microplate wells. Subsequently, 10 µL of the sample was added. The microplate was shaken for 5 s and the absorbance of the reaction was measured at 290 nm for 3 min at 25 °C to determine nonspecific ascorbate degradation. The APx reaction was initiated by adding 20 µL of 50 mM H_2_O_2_. The activity was determined by measuring the decrease in the reaction rate at 290 nm for 15 min. Activity was calculated using the 2.8 mM^−1^ cm^−1^ ascorbate extinction coefficient and expressed in milliunits of enzyme activity per gram of fresh tissue (mU/g FW).

### 4.8. Statistical Analysis

Results from growth and biochemical markers were statistically analyzed using the software PRIMER v6 using pairwise Permutational Multivariate Analysis of Variance (PERMANOVA). The null hypotheses tested were as follows: (i) for each temperature, no significant differences existed among the three inoculation conditions; (ii) for each inoculation condition, no significant differences existed between temperature levels. Significant differences were considered only when *p*-value < 0.05, and are identified in the figures with lowercase letters. After checking variance homogeneity (Levene test) and normality (Kolmogorov–Smirnov test), the interaction between temperature and inoculation was analyzed using two-way ANOVA for growth and biochemical parameters. Post hoc comparisons were performed using Tukey’s test (SPSS version 26.0). Heatmaps representing the lipid concentrations for each condition were constructed using MetaboAnalyst 6.0. Data were normalized to the weight of each replicate and autoscaled by the concentration. The matrix gathering biochemical data per condition was used to perform Principal Component Ordination (PCO). Pearson correlation vectors for biochemical descriptors (correlation > 0.5) were superimposed on the PCO graph, allowing the identification of the descriptors that contributed the most to the differences observed among the conditions tested.

### 4.9. Lipidomics Analysis

#### 4.9.1. Extraction Procedure

The extraction procedure was initiated by adding 1 mL of tert-butyl methyl ether/methanol (3:1) to 5 mg of lyophilized leaf tissue. This mixture was then fortified with 200 pmol of each internal lipid standard (1,2,3-17:0 triacylglycerol, 17:1 lyso phosphatidylethanolamine, 17:1 lyso phosphatidylglycerol, 12:0 ceramide) and vortexed vigorously. Samples were then sonicated for 10 min, and 500 μL of H_2_O/methanol (3:1) was added. After vortexing, samples were centrifuged at 10,000× *g* for 10 min and the upper phase was transferred to a new tube and evaporated under nitrogen. Residues were resuspended in 150 μL methanol and centrifuged at 10,000× *g* for 5 min. Next, 130 μL of the supernatant was transferred to conical vials for injection.

#### 4.9.2. Ultra-Performance Liquid Chromatography–Mass Spectrometry (UPLC-MS)

The analysis consisted of a Waters Acquity UPLC system connected to a Waters LCT Premier orthogonal accelerated time-of-flight mass spectrometer (Waters) operated in both positive and negative electrospray ionization modes. Full scan spectra from 50 to 1500 Da were acquired, and individual spectra were summed to produce data points of 0.2 s each. Mass accuracy and reproducibility were maintained by using an independent reference spray via the LockSpray interference. The analytical column was a 100 × 2.1 mm inner diameter, 1.7 mm C8 Kinetex (Phenomenex). The two mobile phases contained 0.2% formic acid and were phase A: methanol, 1 mM ammonium formate; and phase B: H_2_O, 2 mM ammonium formate. The flow rate was 0.3 mL/min. The gradient of A/B solvents started at 80:20 and changed to 90:10 in 3 min; from 3 to 6 min it remained at 90:10; it then changed to 99:1 until minute 15; remained at 99:1 until minute 18; and finally returned to the initial conditions until minute 20. The column was held at 30 °C.

#### 4.9.3. Untargeted Lipidomic Data Analysis

Each ultra-performance liquid chromatography–mass spectrometry (UPLC-MS) data file was converted to CDF format using the Databridge program of MassLynx (4.1) software. This dataset was then imported into a MATLAB (2023b) computational environment by using the mzcdfread and peaks functions from the MATLAB Bioinformatics Toolbox. Data analysis was performed using the ROIMCR approach [117], which consisted of two different steps: first, mass spectrometry (MS) data compression was performed using the previously described region of interest (ROI) strategy; and second, the application of multivariate curve resolution alternating least squares (MCR-ALS), a chemometric technique used for the resolution of pure elution profiles and the spectra of components from unresolved complex mixtures. This combined approach (ROIMCR) has been successfully used in different untargeted metabolomics investigations of LC-MS data [118]. The newly developed MS-ROI interface [119] was used to process the data. From the relative areas of the MCR-ALS-resolved elution profiles, it is possible to estimate the relative amounts of components in every analyzed sample. Using the resulting MCR-ALS-calculated areas of the different samples for each component, a new data table (data matrix) with the areas of each component in its rows and for each sample in its columns was constructed. These matrices were analyzed using Principal Component Analysis (PCA) and Partial Least Squares Discriminant Analysis (PLS-DA) for each bacterial strain dataset. The Variable Importance in the Projection (VIP) values [120] which estimate the importance of each of the variables (lipids) in the projection used in a PLS model, were calculated to investigate the most influential lipids in the discrimination of samples.

For the identification of lipid compounds, a homemade database of lipids and external databases available online, such as LipidMaps and the Human Metabolome Database, were used. The assigned compound corresponded to the lipid molecule with the minimum mass error value with respect to the measured *m*/*z*, considering the possible adducts in the corresponding ionization modes. The annotated lipid also had to fulfill an adequate retention time regarding its polarity. Fold peak area changes were calculated from the arithmetic mean of the peak area values of each lipidic group. To check whether the differences observed in lipid peak areas between exposed and unexposed samples were statistically significant, Welch’s *t*-test was used. Only identified compounds with VIP values greater than one and statistically significant in Welch’s *t*-test (*p* < 0.05) in at least one of the treatments with respect to the control were finally selected for the hierarchical clustering heatmap analysis and for further interpretation of lipidomic results.

## 5. Conclusions

The world population is expected to increase and, combined with climate change scenarios, will impose pressure on food production. Different models describe an increase in planet surface temperature, and it is essential to ensure important crop outputs, of which *Zea mays* stands out for its economic and social importance. In this study, it was shown that high temperature significantly decreased seed germination; however, at 36 °C, inoculation with the two PGPB strains increased the germination rate. Bacteria promoted plant growth, an effect that was maintained, or increased, in heat stress conditions. Temperature induced molecular and enzymatic antioxidant responses that could not placate oxidative stress or overcome membrane damage of non-inoculated plants. At 36 °C, *Herbaspirillum* sp. inoculation increased GSTs (3-fold) and CAT (+20%) activity, indicating some degree of antioxidant protection conferred by this strain. *Pantoea* sp. inoculation also decreased lipid peroxidation, which was related to the ability of this strain to effectively induce membrane protective mechanisms, such as APx activity (+38%), reducing the need for lipid remodeling and maintaining cell functioning closer to control conditions. Owing to its enhanced protective effects on membranes, *Pantoea* sp.-inoculated plants were selected for further lipidomics analysis. Detailed interpretation of lipid changes evidenced a higher proportion of long-chain saturated lipids, allowing higher lipid packing and an increase in intermolecular forces, resulting in membrane stability. An increase in reserve glycerolipids may indicate lipid membrane remodeling. The galactolipid (mainly MGDG and DGDG) saturation degree and chain length did not change with temperature, pointing to other mechanisms of maintaining photosynthetic membrane integrity, such as active antioxidant mechanisms in chloroplasts, such as APx. The increases in sphingolipid and sterol concentrations at high temperatures are indicative of membrane stability and membrane-associated protein functionality under temperature stress. Overall, both bacterial strains were beneficial to plants exposed to heat stress, with *Pantoea* sp. showing superior performance.

Our study provides novel information on the effects of PGPB inoculation on plant tolerance to heat stress. The influence on lipid remodeling is particularly novel and provides valuable information for biotechnological programs aimed at increasing thermotolerance in plants. This study also underlines the effectiveness of PGPB inoculation as a strategy to increase crop establishment and growth under unfavorable conditions, contributing to sustainable production and food security in increasing temperature scenarios. Further investigations should include different types of soil to more closely simulate the conditions in actual agricultural production and systematically analyze the specific impact of soil type on the experimental results.

## Figures and Tables

**Table 1:** Comparison of key findings from this study and previous research.

Our Results	Previous Results	Previous Studies
The strains were able to increase the dry weight of inoculated *Zea mays* plants in control and heat stress conditions.	Increased yield in temperature-stressed maize plants inoculated with *Paenibacillus alvei*, *Bacillus safensis*, *Bacillus pumilus*, and *Brevundimonas vesicularis.*	Breedt et al. [20]
At 26 °C, only *Herbaspirillum* sp. increased germination, but at 36 °C, both strains increased the germination rate and were able to increase shoot and root growth in *Zea mays.*	Increase in seed germination rate and early development in maize inoculated with strains of *Burkholderia cepacian* and *Burkholderia graminis.*	Dos Santos et al. [23]
Both *Pantoea* sp. and *Herbaspirillum* sp. were able to produce IAA at high temperature, and also increased the germination rate, root elongation, and plant weight in *Zea mays.*	*Aeromonas encheleia* and *Pseudomonas azotoformans* significantly increased maize germination rate, root elongation, and seedling weight due their strong ability to produce indole-3-acetic acid (IAA).	Houida et al. [24]
*Herbaspirillum* sp. inoculation significantly increased photosynthetic pigments, while *Pantoea* sp. did not have a significant effect. This effect was enhanced at high temperature.	PGPB inoculation resulted in increased chlorophyll content.	Rojas et al. [44] and Ren et al. [45]
*Herbaspirillum* sp. significantly increased GSTs and CAT and *Pantoea* sp. significantly increased CAT and APx in heat-stressed plants compared to heat-stressed non-inoculated ones, leading to lower LPO levels.	*Bacillus safensis*, *Ochrobactrium pseudogrignonense*, and *Bacillus cereus* were reported to activate antioxidant signaling in plants under heat stress, leading to a reduction in LPO.	Khan et al. [47]
*Herbaspirillum* sp. was able to significantly increase GST, SOD, and CAT activity and *Pantoea* sp. significantly induced the activity of GSTs, SOD, CAT, and APx at the control temperature.	Some beneficial microorganisms such as *Pseudomonas* strains can enhance the activity of antioxidant enzymes (APX, CAT, SOD) in plants.	González et al. [46]
Neither an increase in saturated fatty acids nor a decrease in unsaturated fatty acids at 36 °C was observed.	Bacteria inoculation on ryegrass increased FA biosynthesis by 65%.	Castanheira et al. [56]
Tocopherols did not change significantly among the conditions tested.	There were reported increases in tocopherols in *Solanum lycopersicum* grown at high temperature.	Spicher et al. [80]
Higher temperature increased sphingolipid (SPB and HexCer) concentration.	It was reported that heat stress led to an additional increase in all sphingolipid subclasses, especially the levels of SPB phosphates and HexCer in tobacco.	Krawkczyk et al. [93]
PI, PC, and PE increased with temperature. *Pantoea* sp. inoculation further increased PI, PC, and PE content.	It was found that PC and PE contents generally decreased in wheat under high temperature stress.	Narayanan et al. [95]
	Heat stress increased PC content with no change in PE levels in leaves of *Atriplex lentiformis*.	Li et al. [98]
No significant changes were observed in PA levels.	Reported an increase in PA levels with temperature in *Zea mays*.	Chen et al. [68]
There was an increase in sterols with temperature. *Pantoea* sp. inoculation further increased sterol content.	Reported increases in sterol levels in *Arabidopsis thaliana* in response to heat stress.	Shiva et al. [51]
	Reported increases in sterol levels in *Withania somnifera* in response to heat stress.	Singh et al. [102]

## Data Availability

The raw data supporting the conclusions of this article will be made available by the authors on request.

## Supplementary Materials

The following supporting information can be downloaded at: https://www.mdpi.com/article/10.3390/plants14162593/s1.
