# Supplementary material for: Maize Crops Under Rising Temperatures: Bacterial Influence on Biochemical and Lipidomic Changes Induced by Heat
Source: Plants (Basel). 2025 Aug 20;14(16):2593. doi: 10.3390/plants14162593 (PMC12389081; doi:10.3390/plants14162593)
Supplement: Supplementary file 1 [file plants-14-02593-s001.zip › plants-3780816-supplementary.pdf]

# Supplementary Materials

**Table S1.** *Zea mays* seed germination as the number of plantlets emerged after 5 days. Results presented for 26 °C and 36 °C and different inoculation conditions (Non-inoculated; *Herbaspirillum* sp. and *Pantoea* sp. Data are means ± SD (n=3). Significant differences ($p < 0.05$) between temperatures for the same inoculation condition are represented with asterisks: (*). Significant differences ($p < 0.05$) among inoculation conditions are represented with uppercase letters for 26 °C and lowercase letters for 36 °C.

| Temperature | Inoculation | Germination (%) |
| --- | --- | --- |
| **26 °C** | Non-inoculated | $93.2 \pm 2.6^{A}$ |
| | *Herbaspirillum* sp. | $96.1 \pm 1.2^{B}$ |
| | *Pantoea sp.* | $93.1 \pm 3.1^{A}$ |
| **36 °C** | Non-inoculated | $57.3 \pm 3.4^{a*}$ |
| | *Herbaspirillum* sp. | $76.3 \pm 2.8^{b*}$ |
| | *Pantoea sp.* | $72.6 \pm 2.4^{b*}$ |

**Table S2.** Colony forming units **(**CFU) counts in soil after *Zea mays* growth influenced by temperature (26 °C and 36 °C) and inoculation (*Herbaspirillum* sp., *Pantoea* sp.). Data are means ± SD (n=3). Significant differences ($p < 0.05$) between temperatures for the same inoculation condition are represented with asterisks: (*). Significant differences ($p < 0.05$) among inoculation conditions are represented with uppercase letters for 26 °C and lowercase letters for 36 °C.

| Temperature | Inoculation | $10^7$ CFU/g soil |
| --- | --- | --- |
| **26 °C** | *Herbaspirillum* sp. | $6.7 \pm 0.7^{A}$ |
| | *Pantoea sp.* | $7.8 \pm 0.8^{A}$ |
| **36 °C** | *Herbaspirillum* sp. | $6.5 \pm 0.4^{a}$ |
| | *Pantoea sp.* | $7.4 \pm 0.3^{b}$ |

**Table S3.** Two-way ANOVA of *Zea mays* growth (root and shoot) and biochemistry parameters.

| Parameter | Factor | MS | F | df | Sig. |
|---|---|---|---|---|---|
| Shoot dry weight | temperature | 0.0 | 5.3 | 1 | 0.0300 |
| | inoculation | 0.0 | 23.5 | 2 | 0.0000 |
| | temperature x inoculation | 0.0 | 4.4 | 2 | 0.0232 |
| Root dry weight | temperature | 0.0 | 0.1 | 1 | ns |
| | inoculation | 0.1 | 38.6 | 2 | 0.0000 |
| | temperature x inoculation | 0.1 | 28.5 | 2 | 0.0000 |
| Protein | temperature | 6586.7 | 45.6 | 1 | 0.0000 |
| | inoculation | 1778.6 | 12.3 | 2 | 0.0002 |
| | temperature x inoculation | 244.6 | 1.7 | 2 | ns |
| ETS | temperature | 0.1 | 135.8 | 1 | 0.0000 |
| | inoculation | 0.0 | 21.4 | 2 | 0.0000 |
| | temperature x inoculation | 0.0 | 14.0 | 2 | 0.0001 |
| Chlorophyll a | temperature | 11113.4 | 3.7 | 1 | ns |
| | inoculation | 89748.8 | 29.9 | 2 | 0.0000 |
| | temperature x inoculation | 4539.9 | 1.5 | 2 | ns |
| Chlorophyll b | temperature | 11615.8 | 14.3 | 1 | 0.0009 |
| | inoculation | 75017.2 | 92.4 | 2 | 0.0000 |
| | temperature x inoculation | 5651.5 | 7.0 | 2 | 0.0041 |
| Carotenoids | temperature | 0.0 | 0.0 | 1 | ns |
| | inoculation | 508.0 | 3.8 | 2 | 0.0376 |
| | temperature x inoculation | 2841.8 | 21.1 | 2 | 0.0000 |
| Starch | temperature | 0.3 | 0.4 | 1 | ns |
| | inoculation | 15.9 | 22.0 | 2 | 0.0000 |
| | temperature x inoculation | 5.4 | 7.5 | 2 | 0.0030 |
| Soluble carbohydrates | temperature | 11.7 | 14.9 | 1 | 0.0007 |
| | inoculation | 7.6 | 9.8 | 2 | 0.0008 |
| | temperature x inoculation | 6.2 | 7.9 | 2 | 0.0023 |
| Proline | temperature | 43319.4 | 351.2 | 1 | 0.0000 |
| | inoculation | 11050.9 | 89.6 | 2 | 0.0000 |
| | temperature x inoculation | 7411.2 | 60.1 | 2 | 0.0000 |
| GSTs | temperature | 112.3 | 11.6 | 1 | 0.0023 |
| | inoculation | 4761.7 | 490.6 | 2 | 0.0000 |
| | temperature x inoculation | 752.8 | 77.6 | 2 | 0.0000 |
| SOD | temperature | 409.3 | 10.4 | 1 | 0.0037 |
| | inoculation | 285.2 | 7.2 | 2 | 0.0035 |
| | temperature x inoculation | 3038.1 | 76.9 | 2 | 0.0000 |
| CAT | temperature | 1623.1 | 77.5 | 1 | 0.0000 |
| | inoculation | 506.9 | 24.2 | 2 | 0.0000 |
| | temperature x inoculation | 38.4 | 1.8 | 2 | ns |
| GPX | temperature | 266856.9 | 148.5 | 1 | 0.0000 |
| | inoculation | 7768.3 | 4.3 | 2 | 0.0249 |
| | temperature x inoculation | 29791.8 | 16.6 | 2 | 0.0000 |
| APX | temperature | 22.2 | 71.6 | 1 | 0.0000 |
| | inoculation | 13.5 | 43.6 | 2 | 0.0000 |
| | temperature x inoculation | 1.1 | 3.4 | 2 | 0.0494 |
| LPO | temperature | 0.0 | 1.1 | 1 | ns |
| | inoculation | 1.4 | 33.9 | 2 | 0.0000 |
| | temperature x inoculation | 0.9 | 21.1 | 2 | 0.0000 |
| Protein carbonylation | temperature | 7.9 | 46.7 | 1 | 0.0000 |
| | inoculation | 1.4 | 8.0 | 2 | 0.0022 |
| | temperature x inoculation | 0.5 | 2.8 | 2 | ns |
